# A Comparison of Methods for Tracking Muscle Quality During Early-Phase Rehabilitation Following Anterior Cruciate Ligament Reconstruction

**DOI:** 10.3390/jfmk11020200

**Published:** 2026-05-17

**Authors:** Matt S. Stock, Heather N. Fowler, Ashleigh L. Ditmyer, Charles E. Nyberg, Debbie L. Hahs-Vaughn, Randi M. Richardson

**Affiliations:** 1 Cognition, Neuroplasticity, and Sarcopenia (CNS) Laboratory, Institute of Exercise Physiology and Rehabilitation Science, University of Central Florida, Orlando, FL 32816, USA; 2 Department of Learning Sciences and Educational Research, University of Central Florida, Orlando, FL 32816, USA

**Keywords:** ACL, knee injury, echo intensity, ultrasound imaging, muscle atrophy

## Abstract

**Background:** Echo intensity (EI) has emerged as a promising and accessible tool for tracking changes in skeletal muscle quality; however, its utility during early-phase rehabilitation has not been studied. Using an observational cohort design, we examined changes in quadriceps muscle strength, size, and quality, along with self-reported knee function, 2, 6, and/or 10 weeks following anterior cruciate ligament reconstruction (ACLR). **Methods**: Thirteen participants (4 males, 9 females; mean age = 23 years) were assessed for bilateral isometric peak torque and cross-sectional area (CSA) and corrected EI of the vastus lateralis and rectus femoris. Self-reported knee function was measured using the International Knee Documentation Committee (IKDC) questionnaire. **Results**: Quadriceps peak torque was significantly lower in the surgical limb at 2 weeks following surgery but increased from weeks 2 to 10, while the nonsurgical limb remained stable. IKDC scores improved significantly over time. Vastus lateralis CSA decreased in the surgical limb between weeks 2 and 6, while rectus femoris CSA increased between weeks 6 and 10 in both limbs. Corrected EI values did not change over time. No significant correlations were observed among changes in muscle strength, size, quality, or self-reported knee function. **Conclusions**: We conclude that quadriceps strength, size, quality, and self-reported knee function change independently and do not follow a shared recovery trajectory.

## 1. Introduction

Anterior cruciate ligament (ACL) injury is among the most common orthopedic conditions, affecting about 70 people out of every 100,000 annually [1]. From 2010 to 2020, ACL injuries modestly declined overall, although ACL reconstruction became relatively more common over time, particularly among adolescents and females [2]. Muscle weakness and atrophy are two expected outcomes following ACL reconstruction (ACLR) surgery that negatively impact physical function and delay a patient’s return to activity [3,4]. Despite advances in ACLR research and dedicated clinical efforts, rehabilitation outcomes remain suboptimal. Recent studies indicate that only 65% of patients return to their pre-injury level of function, with persistent quadriceps weakness commonly identified as a limiting factor [5]. Therefore, research focused on improving the assessment and tracking of rehabilitation progress is critically important to advancing patient care.

Quadriceps strength is a well-established determinant of functional recovery following ACLR and has been linked to both objective performance measures and patient-reported outcomes [6]. Similarly, quadriceps muscle atrophy occurs rapidly following surgery due to a combination of disuse [7] and neuromuscular inhibition [8], and may persist for months despite structured rehabilitation [9]. While it is often assumed that changes in muscle strength and size align with patient perceptions of physical function, these variables may follow distinct recovery trajectories. Notably, muscle size and strength alone may not fully account for individual differences in recovery. For example, patients with similar quadriceps peak torque or cross-sectional area (CSA) may report markedly different functional outcomes, highlighting limitations in traditional assessment approaches. Traditional assessments of quadriceps strength and size also do not account for muscle composition, or quality, which has gained increasing attention for its potential to provide unique insights into skeletal muscle changes over time among clinical and aging populations [10,11,12].

Echo intensity (EI), obtained from B-mode ultrasound imaging, is a practical and portable method for estimating skeletal muscle quality by quantifying the amount of non-contractile tissue—such as intramuscular fat [13] and fibrous tissue [14]—within a defined region of interest. EI assessments are relatively quick and easy to implement in clinical settings, and can be reliably performed by novice users after minimal training [15]. Unlike measures of muscle size alone, EI provides additional insight into muscle composition and has been linked to strength [16], disuse [11], and aging-related decline [10]. Although EI has been studied extensively in older adults and clinical populations, its application following ACLR is more recent. Garcia et al. assessed rectus femoris morphology among ACLR patients and found that increased intramuscular fat content was associated with poorer patient-reported knee function [17]. Notably, quadriceps muscle quality remained impaired at return to activity, despite completion of rehabilitation [17]. Similarly, Johnson et al. reported elevated EI values in both the vastus lateralis and rectus femoris of the ACLR limb at one and three months post-surgery, indicating persistent reductions in muscle quality during early recovery [18]. Interestingly, changes in EI and CSA did not occur in parallel, suggesting that early hypertrophy may not reflect improvements in contractile tissue. Supporting the validity of EI as a surrogate for intramuscular fat, Grozier et al. demonstrated a strong relationship between ultrasound-derived EI and MRI-based measurements of fat infiltration in the rectus femoris among young adults 1–5 years post-ACLR [19]. Collectively, these initial studies support the value of EI as a clinically feasible and physiologically meaningful marker of muscle quality in ACLR populations, with implications for both monitoring recovery and guiding rehabilitation progression.

Despite growing interest in tracking quadriceps function following ACLR, key knowledge gaps remain. Comprehensive data on how quadriceps EI changes during rehabilitation and whether these changes align with muscle strength, size, or patient-reported function are lacking. Clarifying how these measures evolve can help clinicians refine and personalize treatment plans. In particular, little is known about muscle recovery during the early rehabilitation phase, which may be critical for long-term success. This study aimed to track changes in muscle strength (peak torque), size (CSA), quality (EI), and self-reported knee function following ACLR. Based on prior research, we hypothesized that strength, EI, and IKDC scores would improve but follow trajectories distinct from CSA.

## 2. Materials and Methods

### 2.1. Study Design

This study utilized a repeated measures design in individuals aged 15–40 years who underwent ACLR within 12 months of injury. To maximize external validity and facilitate implementation in clinical settings, we selected methodological approaches that could be replicated by rehabilitation professionals. All testing equipment was portable, easy to use, and relatively inexpensive. To enhance participant retention, testing was offered either at the University of Central Florida (Orlando, FL, USA) or at the participant’s physical therapy clinic. Each testing session lasted approximately 45 min. The study was designed to assess outcomes at 2, 6, and 10 weeks following ACLR. However, to minimize participant burden and reduce hesitancy, the 2-week visit was made optional. As a result, while some participants completed testing at 2, 6, and 10 weeks, others only completed the 6- and 10-week sessions. This flexible approach was intentionally adopted to support retention, and our robust statistical plan was designed to accommodate this variation in data collection (see below). To minimize diurnal variation, sessions were scheduled at the same time of day (±1 h) and were held either prior to a physical therapy session or at another time that did not follow a therapy session.

During the first visit, participants completed a health intake form that collected medical history information. Each testing session included completion of the International Knee Documentation Committee (IKDC) Questionnaire, as well as assessments of vastus lateralis and rectus femoris CSA and EI and maximal quadriceps peak torque. These assessments are described in detail below in the order they were performed. All study procedures were approved by the University of Central Florida Institutional Review Board (#6096), and all participants provided written informed consent. For participants under the age of 18, parental consent and participant assent were obtained. Data were collected and managed using REDCap electronic data capture tools (Vanderbilt University, Nashville, TN, USA) and was stored on Microsoft Teams (Microsoft Corporation, Redmond, WA, USA). Additional details concerning our study design are described in a Strengthening the Reporting of Observational studies in Epidemiology (STROBE) checklist provided in Supplementary Materials.

### 2.2. Participants

We recruited participants using a combination of convenience and snowball sampling, leveraging clinical partnerships with physical therapists to promote the study. The final sample included 13 participants (9 females, 4 males), ranging from 16 to 35 years of age (mean ± SD: 23 ± 5 years) with a body mass index (BMI) of 25.2 ± 2.9 kg/m^2^. All participants had undergone ACLR within the six weeks prior to enrollment. Before enrolling, participants completed a phone-based pre-enrollment health screening to ensure eligibility and minimize risk. Exclusion criteria included a BMI < 20 or >35 kg/m^2^, significant pathology or pain affecting the nonsurgical limb, bilateral ACLR, or limb amputation. Additional exclusions included current pregnancy, use of hormone therapy in the previous six months, history of intra-articular injections, cancer, stroke, or any metabolic, neuromuscular, or degenerative disease. English proficiency was required.

The final cohort included five quadriceps tendon autografts, six bone-patellar tendon-bone autografts, and one hamstring tendon autograft. Six of the 13 participants reported needing meniscus repair in conjunction with ACLR. One participant reported a prior (non-index) meniscal surgery. Post-operative management varied, with weight-bearing status ranging from non-weight bearing to weight bearing as tolerated, and crutch use ranging from 3 to 21 days (mean ~10 days). No participants reported concomitant lateral augmentation. More detailed surgical variables (e.g., fixation methods, graft construct specifics) were not collected. It is important to note that surgery data were based on self-reports and not verified through operative reports; this represents an important limitation of the study.

All participants were engaged in physical therapy two to three times per week and were prescribed a home exercise program; however, as assessing the efficacy of rehabilitation was not a study aim, formal data were not collected regarding session content, frequency, or adherence.

### 2.3. Ultrasound Image Acquisition and Analysis

**Figure 1:**
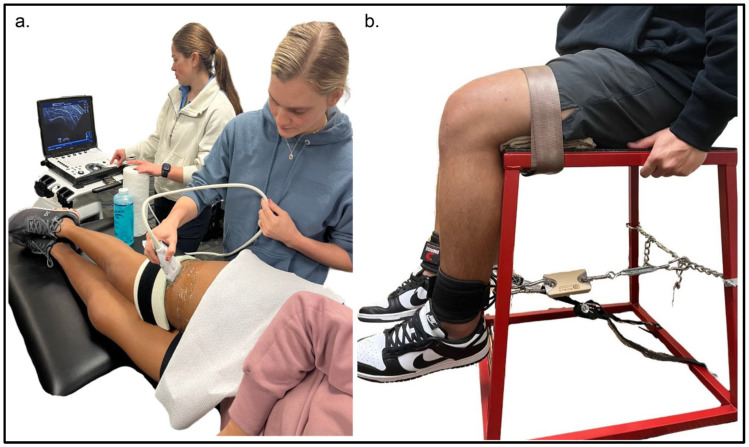
Visual depiction of our methods used to acquire B-mode ultrasound images from the quadriceps (vastus lateralis) (**a**) and our custom setup used to assess isometric peak torque with a Tindeq dynamometer (**b**). For strength testing, participants were positioned in a seated posture with the hip and knee at 90° flexion. A strap secured around the distal tibia was connected to the Tindeq dynamometer to measure maximal voluntary isometric contractions of the quadriceps. This configuration enabled consistent and reproducible torque assessment across testing sessions.

B-mode ultrasonography was used to assess the vastus lateralis and rectus femoris muscles in the transverse plane using a portable ultrasound device (Logiq e BT12, GE Healthcare, Milwaukee, WI, USA) equipped with a multi-frequency linear-array probe (12 L-RS, 5–13 MHz, 38.4 mm field of view). The same well-trained investigator acquired all ultrasound images. Measurements for both muscles were obtained at 50% of their respective muscle lengths (Figure 1a). For the rectus femoris, this corresponded to halfway between the anterior inferior iliac spine and the superior border of the patella, whereas the site for the vastus lateralis was halfway between the greater trochanter and the superior border of the patella. A high-density foam pad was gently strapped over the participant’s leg to ensure consistent, linear movement of the probe in the transverse plane. A generous amount of water-soluble transmission gel (Aquasonic 100, Parker Laboratories, Inc., Fairfield, NJ, USA) was applied to optimize acoustic coupling. For each muscle, three transverse-plane images were collected bilaterally, and the clearest image for each muscle at each visit was selected for analysis. We began by capturing images of the non-surgical rectus femoris in the supine position, followed by the non-surgical vastus lateralis in the side-lying position (knee positioned in 15 degrees of flexion). Most ultrasound settings (Frequency: 10 MHz, Gain: 55 dB, Dynamic Range: 72) were held constant. An image depth of 5.0 cm was used for all but 2 participants, who required image depths of 4.0 and 6.0 cm; however, previous work has shown minimal influence of image depth on EI [20].

Selected ultrasound images were analyzed using ImageJ software (version 1.51, National Institutes of Health, Bethesda, MD, USA). Using the polygon function, the visible muscle borders were manually outlined to define the region of interest while carefully excluding surrounding fascia. EI was quantified via grayscale histogram analysis, producing values from 0 (black) to 255 (white) in arbitrary units (A.U.). Subcutaneous tissue thickness (cm) was measured at three locations (left, center, right) within each image using the straight-line function to assess the distance from the skin–muscle interface to the superficial aponeurosis. These values were averaged and used to correct raw EI values using the equation developed by Young et al.: Corrected EI = Raw EI + [40.5278 × subcutaneous fat thickness (cm)] [13]. CSA was calculated using the area function in ImageJ. Test–retest reliability for these measurements has been reported previously [15]. The same investigator conducted all image analyses; they were not blinded to limb or time point. Excellent test–retest reliability of a subset of ultrasound images was demonstrated for all ultrasound outcomes and both muscles (intraclass correlation coefficients > 0.95, standard errors of measurement < 5.0%).

### 2.4. IKDC

At each visit, participants completed the IKDC questionnaire to assess their self-reported level of knee function. The IKDC is a validated outcome measure designed to evaluate symptoms, function, and sports activity in individuals with a variety of knee conditions, including those recovering from ACLR [21,22]. It consists of 18 items that assess domains such as pain, stiffness, swelling, activities of daily living, and ability to perform sports-related movements. Each item is scored, and responses are transformed to a scale ranging from 0 to 100, with higher scores indicating better perceived knee function. The questionnaire was administered digitally using an iPad or laptop. Scoring was performed according to standardized IKDC guidelines [21], and the resulting numerical scores were used in subsequent data analyses.

### 2.5. Assessment of Maximal Isometric Torque

Quadriceps isometric torque testing was performed using a custom-designed setup with participants seated on a plyometric box (Figure 1b). Force data were acquired using a Tindeq Progressor 200 Bluetooth dynamometer (Tindeq AS, Leknes, Norway), a portable and reliable device that is increasingly being utilized by clinicians to assess muscle function [23]. Each participant was positioned with the hip and knee flexed to 90°, and the setup was adjusted individually to ensure consistent joint angles across participants. This joint position was selected because participants were assessed approximately two weeks following ACL reconstruction, when patient safety and graft protection were important considerations. Testing at 90° of knee flexion is generally well tolerated during the early postoperative period and minimizes anterior tibial shear forces compared with more extended knee positions, thereby allowing for safe assessment of maximal voluntary quadriceps torque. The ankle cuff and dynamometer strap were secured and aligned to maintain the 90° knee angle. A belt strap was fastened across the participant’s lap to help stabilize the hips and maintain the desired posture throughout testing. Data were collected in the same order for each participant, starting with the non-surgical limb prior to the surgical limb. Prior to testing, participants performed a submaximal warm-up by gently performing five 3 s isometric knee extensions at progressively greater ratings of perceived maximum effort (i.e., 50%, 60%, 70%, 80%, 90% of perceived maximum). Following the warm-up, the participants performed 5 maximal voluntary isometric contractions lasting no more than 3 s separated by a minimum of 30 s rest between attempts. During each trial, participants were given the verbal cue of “push hard and fast”. They were provided with visual feedback of their force output in real time. Data were collected in real time using the Tindeq mobile application installed on an iPad, allowing for immediate visualization and export of peak force values. The highest peak force from the 5 attempts was recorded and utilized for our analysis. To convert force (N) to torque (Nm), shank length was measured as the distance from the lateral femoral epicondyle to the center of the ankle cuff attachment point.

### 2.6. Statistical Analysis

A two-level multilevel increase model using restricted maximum likelihood was used to estimate fixed and random effects with the lme4 package in R [24]. Tests of model fit were calculated using estimates from full information maximum likelihood. Repeated measures (i.e., level one, N = 31 for all outcomes except surgical and non-surgical peak torque with N = 29) were nested within participants (i.e., level two, N = 13). Multilevel models have several advantages including providing more accurate estimates of standard errors given the dependency of repeated measures within person which leads to more accurate predictions and the ability to analyze all available data even if participants were not measured on all occasions, among other advantages.

The intraclass correlation coefficient (ICC) was estimated from an unconditional means model and generally ranged from 0.12 to 0.97, providing support to warrant multilevel modeling. The final model was a random intercepts model which included time-invariant predictors (sex, entered uncentered (male = 0; female = 1); graft–hamstrings and graft–quadriceps, entered uncentered (reference was graft–patellar); and BMI and age, both centered at the mean for the sex of the participant). Statistical significance was set at *p* < 0.05.

## 3. Results

### 3.1. Multilevel Results

Of the 13 participants, one completed the 2- and 6-week visits only, six completed the 6- and 10-week visits only, and six completed all three testing sessions (2, 6, and 10 weeks). Reasons for non-participation included unanticipated travel and learning of the study following the 2-week time point. Table 1 presents select multilevel statistics. The intercept represents the average outcome at two weeks post-surgery. The slopes represent the weekly change in the outcome from two to six weeks or six to 10 weeks, respectively. Confidence intervals of the unstandardized regression coefficients were reviewed to compare change over time for surgical and nonsurgical limbs. Non-overlapping confidence intervals suggest statistically significant differences [25]. The final models were evaluated to assess whether key multilevel assumptions were met, including linearity, normally distributed and equal variances of level-one residuals, and multivariate normality and homoscedasticity of level-two residuals. Shapiro–Wilk’s and Mardia’s tests indicated that the normality assumptions at both levels were generally acceptable. Levene’s test (centered on the median) supported the assumption of equal variances at level one. Additionally, scatterplots of residuals versus predictors suggested that the assumptions of linearity and homoscedasticity were reasonably met.

### 3.2. Peak Torque

**Figure 2:**
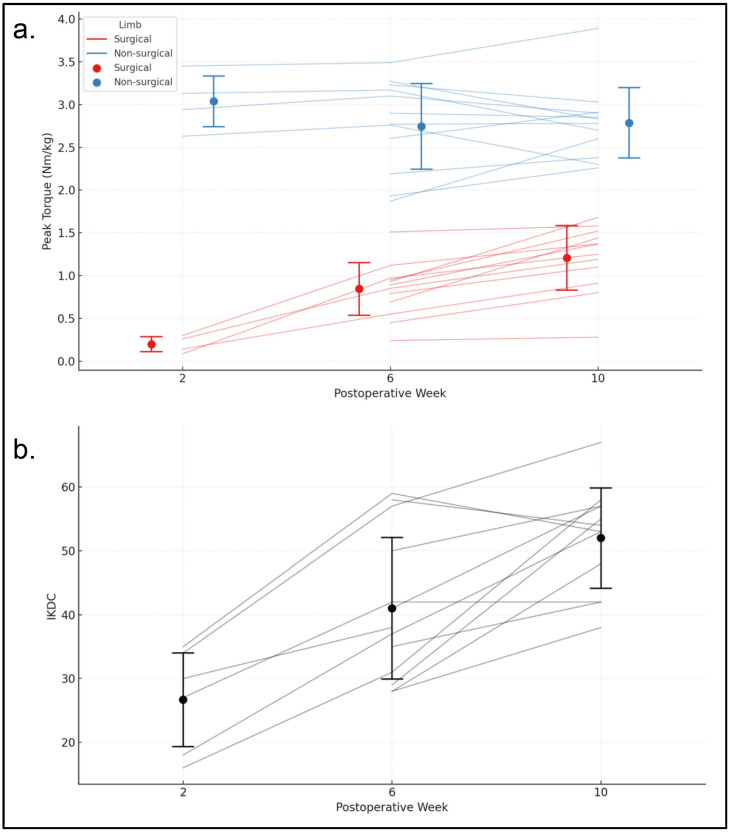
(**a**) Isometric peak torque (Nm/kg) for the surgical and non-surgical limbs at 2, 6, and 10 weeks following ACLR. Thin blue lines represent individual trajectories for the surgical limb, while red lines represent the non-surgical limb. Circles indicate group means, and error bars represent standard deviations. (**b**) IKDC scores for each participant and the group across the 2-, 6-, and 10-week timepoints.

At two weeks post-surgery, the mean peak torque was 0.19 Nm/kg for the surgical limb and 3.17 Nm/kg for the nonsurgical limb (Figure 2a). The surgical limb showed a statistically significant increase in peak torque between weeks 2–6 and 6–10, with average weekly gains of 0.16 and 0.05 Nm/kg, respectively. In contrast, peak torque did not improve over time in the nonsurgical limb. Sex, BMI, age, and graft type were not significant predictors of peak torque for either limb. Confidence intervals indicated that, at two weeks post-surgery, the nonsurgical limb had significantly greater peak torque than the surgical limb. However, the surgical limb demonstrated significantly greater improvements between weeks 2–6 and 6–10. The relationships between peak torque and the predictor variables were similar for both limbs.

### 3.3. IKDC

The average IKDC score at two weeks post-surgery was 25.30 (Figure 2b). There was a statistically significant increase in IKDC scores over time, with weekly gains of 4.12 units between weeks 2 and 6 and 1.39 units between weeks 6 and 10. Sex, BMI, age, and graft type were not significant predictors of IKDC scores.

### 3.4. Vastus Lateralis CSA

**Figure 3:**
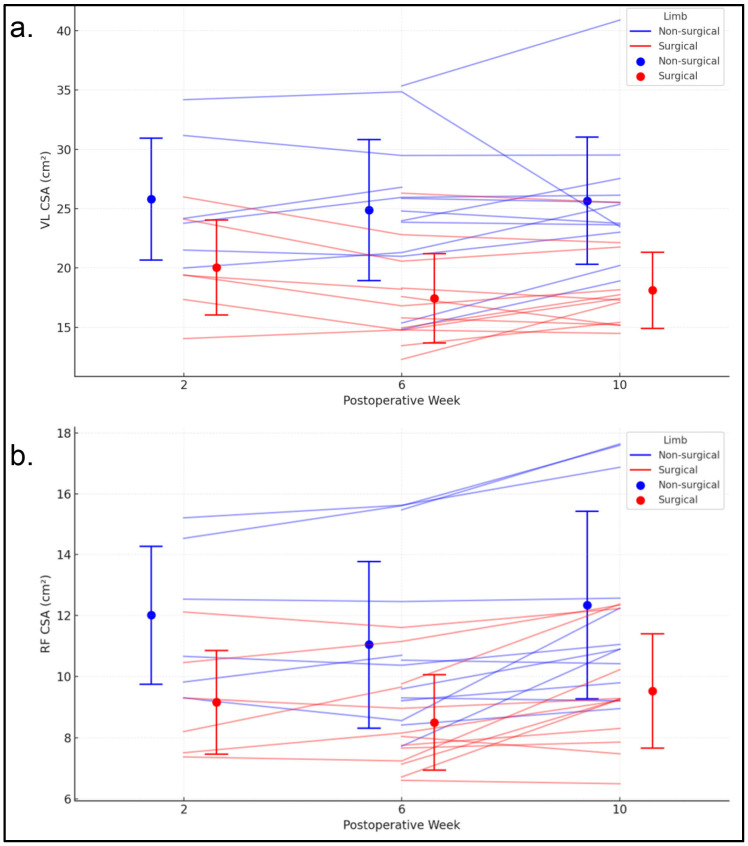
(**a**) CSA of the vastus lateralis for the surgical and non-surgical limbs at 2, 6, and 10 weeks following ACLR. Thin blue lines represent individual trajectories for the surgical limb, while red lines represent the non-surgical limb. Circles indicate group means, and error bars represent SD. (**b**) CSA of the rectus femoris across the same timepoints, with individual and group responses displayed as in panel (**a**). VL = vastus lateralis; RF = rectus femoris.

At two weeks post-surgery, the average vastus lateralis CSA was similar between the surgical limb (20.94 cm^2^) and the nonsurgical limb (26.98 cm^2^) (Figure 3a). A statistically significant decrease in vastus lateralis CSA was observed in the surgical limb between weeks 2 and 6, while no significant changes occurred in either limb between weeks 6 and 10. For the surgical limb, vastus lateralis CSA increased by approximately 0.57 cm^2^ for each additional year of age. Sex, BMI, and graft type were not significant predictors for either limb. Based on confidence intervals, the mean vastus lateralis CSA at two weeks, changes over time, and relationships with predictors were generally similar between the surgical and nonsurgical limbs.

### 3.5. Rectus Femoris CSA

At two weeks post-surgery, the average rectus femoris CSA was 8.74 cm^2^ for the surgical limb and 11.27 cm^2^ for the nonsurgical limb (Figure 3b). There was no significant change in rectus femoris CSA between weeks 2 and 6; however, a significant increase was observed between weeks 6 and 10, with weekly gains of 0.13 cm^2^ for the surgical limb and 0.16 cm^2^ for the nonsurgical limb. Sex, BMI, age, and graft type were not statistically significant predictors of rectus femoris CSA for either limb. Confidence intervals indicated that the mean rectus femoris CSA at two weeks, changes over time, and relationships with predictors were generally similar between the surgical and nonsurgical limbs.

### 3.6. Vastus Lateralis EI

**Figure 4:**
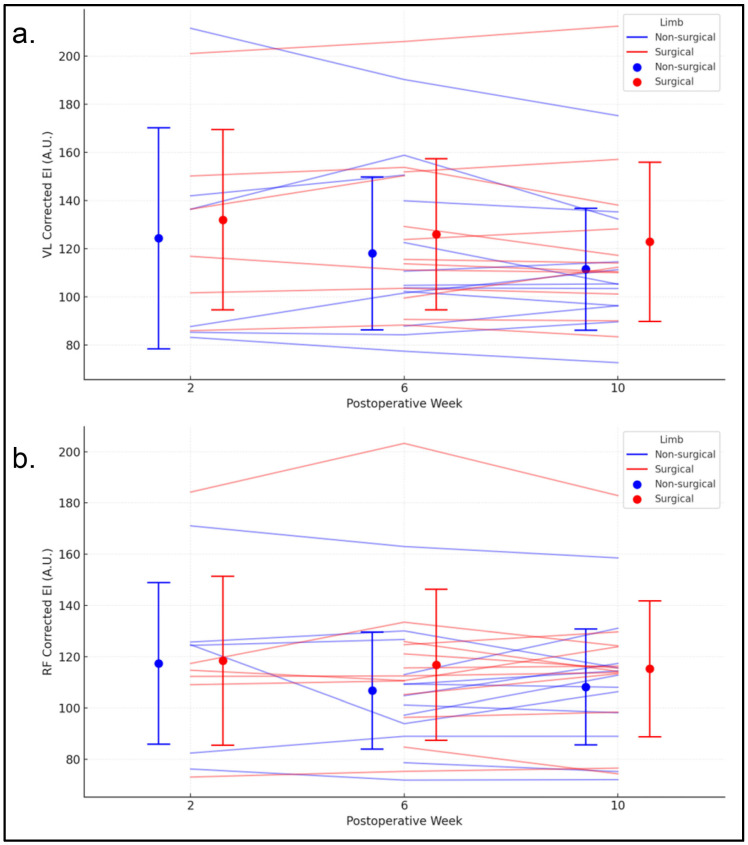
(**a**) Corrected EI of the vastus lateralis for the surgical and non-surgical limbs at 2, 6, and 10 weeks following ACLR. Thin blue lines represent individual trajectories for the surgical limb, while red lines represent the non-surgical limb. Circles indicate group means, and error bars represent SD. (**b**) Corrected EI of the rectus femoris across the same timepoints, with individual and group responses displayed as in panel (**a**). VL = vastus lateralis; RF = rectus femoris.

At two weeks post-surgery, the average corrected echo intensity of the vastus lateralis was similar between the surgical limb (119.23 A.U.) and the nonsurgical limb (108.13 A.U.) (Figure 4a). BMI was positively associated with corrected echo intensity, with statistically significant increases of 8.85 A.U. for the surgical limb and 3.75 A.U. for the nonsurgical limb for each one-unit increase in BMI. There were no statistically significant changes in corrected echo intensity over time for either limb. Age was positively associated with corrected echo intensity in the nonsurgical limb, increasing by 3.75 units for each additional year of age. Sex and graft type were not statistically significant predictors for either limb. Based on the confidence intervals, the mean corrected echo intensity at two weeks post-surgery, changes over time, and relationships with predictors were generally similar between the surgical and nonsurgical limbs.

### 3.7. Rectus Femoris EI

At two weeks post-surgery, the average corrected EI of the rectus femoris was similar between the surgical limb (107.11 A.U.) and the nonsurgical limb (110.29 A.U.) (Figure 4b). There were no statistically significant changes in corrected EI over time for either limb. BMI was a significant predictor in both limbs, with increases of approximately 9.05 units in the surgical limb and 12.65 units in the nonsurgical limb for each one-unit increase in BMI. Sex, age, and graft type were not statistically significant predictors for either limb. Based on the confidence intervals, the mean corrected EI at two weeks post-surgery, changes over time, and relationships with predictors were generally similar between the surgical and nonsurgical limbs.

### 3.8. Correlations Among Change Scores

To further explore relationships among the recovery domains, bivariate correlations were performed using change scores between weeks 6 and 10 for the surgical limb (N = 12). Changes in IKDC scores were not associated with changes in peak torque (r = 0.03, *p* = 0.915), CSA, or corrected EI. However, a moderate negative correlation between IKDC and vastus lateralis CSA approached significance (r = –0.57, *p* = 0.053), suggesting that individuals reporting greater functional improvement tended to experience smaller increases—or greater decreases—in vastus lateralis CSA. This finding, while counterintuitive, may reflect early neural or perceptual recovery outpacing measurable hypertrophy in some participants. Peak torque change scores showed a moderate, nonsignificant correlation with rectus femoris CSA (r = 0.41, *p* = 0.190), but were otherwise weakly associated with vastus lateralis CSA and corrected EI (Table 2).

## 4. Discussion

We characterized longitudinal changes in quadriceps strength, size, quality, and self-reported function during the first 10 weeks following ACLR. As expected, the surgical limb exhibited substantial early impairments. Peak torque increased by over 500% from weeks 2 to 10, with the largest gains between weeks 2 and 6, suggesting rapid neuromuscular improvements early in rehabilitation. IKDC improved by 95% over the same period, with the steepest gains also observed in the first six weeks. In contrast, vastus lateralis CSA declined by 13.1% from weeks 2 to 6 and recovered slightly by week 10. Rectus femoris CSA showed a similar pattern, with only modest gains by week 10. Corrected EI for both vastus lateralis and rectus femoris remained largely unchanged over time. An exploratory correlation analysis for 6-to-10 week data revealed no strong associations among changes in peak torque, IKDC, CSA, and EI. These findings highlight a clear disparity across outcomes. While strength and self-reported function improved substantially during the first ten weeks post-ACLR, CSA and EI demonstrated minimal or no recovery, emphasizing the asynchronous and domain-specific nature of neuromuscular, morphological, and perceptual recovery early after surgery.

While EI has received growing attention as a non-invasive marker of muscle quality, our findings suggest that it may have limited sensitivity during the early phase of ACLR rehabilitation. Corrected EI values for both the vastus lateralis and rectus femoris did not change significantly over the 10-week period, despite substantial improvements in torque and IKDC scores. These results contrast with the common assumption that muscle composition improves alongside function and strength during recovery. Johnson et al. reported elevated EI values in both the vastus lateralis and rectus femoris at one and three months post-ACLR, with no significant change over time, supporting the notion that EI may remain stable even as other domains improve [18]. Similarly, Garcia et al. observed that muscle quality, measured via percent fat in the RF, was associated with IKDC scores early after surgery, whereas CSA alone was not [4]. In our study, EI was not significantly correlated with strength or function, raising questions about its responsiveness to short-term rehabilitation and whether it captures relevant biological changes during this critical window.

One explanation for our findings is that EI better reflects long-term changes in intramuscular composition rather than rapid adaptations. Grozier et al. recently validated EI as a surrogate for MRI-derived intramuscular fat in ACLR patients 1–5 years post-surgery, providing support for its use in assessing chronic deficits [19]. However, our findings suggest that during the first 10 weeks after ACLR, EI may lack the temporal resolution needed to detect early remodeling of muscle tissue. This is further supported by the work of Fasih et al. [26], who identified substantial and persistent asymmetries in EI and muscle architecture between limbs years after surgery. Together, these studies imply that while EI is valuable for identifying residual deficits in chronic stages of recovery, it may not be suitable as an early-phase outcome measure. The disconnect between functional gains and unchanged EI values underscores the complexity of neuromuscular recovery and highlights the need to use EI cautiously when evaluating short-term rehabilitation efficacy.

This study has several strengths, including a pragmatic design intended to enhance external validity, assessment of multiple quadriceps muscles, and inclusion of both objective and patient-reported outcomes. Nevertheless, several limitations should be acknowledged. First, the relatively small sample size (N = 13, with one participant missing data for selected analyses) may have reduced our ability to detect modest associations or temporal changes, particularly for echo intensity, which is characterized by substantial inter-individual variability. Accordingly, the absence of statistically significant changes in echo intensity should be interpreted cautiously, and these findings should be considered exploratory and hypothesis-generating rather than definitive evidence of no effect. Second, although the Tindeq dynamometer provides a portable and clinically feasible method for assessing isometric knee extension torque, it has not been as extensively validated as traditional isokinetic dynamometry and may yield different absolute strength estimates. Third, because participants were first assessed approximately two weeks following ACL reconstruction, preoperative measurements were not available, limiting our ability to quantify the magnitude of change relative to each participant’s baseline status. Additionally, several sport- and clinically related factors that may influence early recovery were not systematically collected, including sport type, competitive level, training volume, pain, joint effusion, range of motion limitations, bracing status, and weight-bearing restrictions. Although all participants were engaged in outpatient rehabilitation and assessed at standardized post-operative time points (2, 6, and 10 weeks), we did not track the specific content, frequency, or adherence to rehabilitation, which may have contributed to variability in recovery trajectories. The broad and heterogeneous nature of the cohort was intentional and reflective of a real-world clinical population; however, this pragmatic approach may have increased variability and limited our ability to isolate the influence of specific clinical or sport-related factors. Future studies with larger sample sizes, standardized rehabilitation protocols, more comprehensive clinical characterization, and longer-term follow-up are needed to better define the responsiveness of ultrasound-derived measures of muscle quality throughout recovery following ACL reconstruction.

In summary, this study tracked changes in quadriceps strength, size, muscle quality, and self-reported function during the first 10 weeks of rehabilitation following ACLR. Participants showed substantial improvements in peak torque and IKDC scores, particularly between weeks 2 and 6. However, CSA demonstrated only modest recovery, and EI did not significantly change over time. No significant associations were observed among the measured outcomes, suggesting that each domain reflects a distinct aspect of recovery. These findings underscore the need for a multifaceted approach to monitoring early rehabilitation progress after ACLR.

## Figures and Tables

**Table 1:** Regression coefficients (*p* values) and confidence intervals (CIs) for the final model. IKDC = International Knee Documentation Committee; CSA = cross-sectional area; EI = echo intensity.

Outcome	2 Weeks (Intercept)	2 Weeks *CI*	2-to-6 Weeks (Slope)	2-to-6 Weeks Slope *CI*	6-to-10 Weeks (Slope)	6-to-10 Weeks Slope *CI*
IKDC	25.30 (0.004)	14.19, 36.93	4.12 (<0.001)	2.23, 5.79	1.39 (0.001)	0.71, 2.08
Surgical Peak Torque	0.19 (0.400)	−0.15, 0.52	0.16 (<0.001)	0.11, 0.21	0.05 (<0.001)	0.03, 0.06
Nonsurgical Peak Torque	3.17 (<0.001)	2.77, 3.67	−0.02 (0.580)	−0.14, 0.04	0.003 (0.820)	−0.02, 0.03
Surgical Vastus Lateralis CSA	20.95 (<0.001)	17.91, 23.98	−0.50 (0.001)	−0.85, −0.14	0.09 (0.220)	−0.05, 0.22
Nonsurgical Vastus Lateralis CSA	26.98 (<0.001)	22.02, 31.71	0.02 (0.960)	−0.70, 0.83	0.11 (0.490)	−0.19, 0.40
Surgical Rectus Femoris CSA	8.74 (<0.001)	6.77, 10.75	0.07 (0.550)	−0.16, 0.26	0.13 (0.004)	0.06, 0.21
Nonsurgical Rectus Femoris CSA	11.28 (<0.001)	8.21, 14.33	0.07 (0.510)	−0.15, 0.28	0.16 (<0.001)	0.08, 0.24
Surgical Vastus Lateralis EI	119.23 (<0.001)	101.10, 137.50	0.49 (0.530)	−1.04, 1.89	−0.21 (0.470)	−0.77, 0.33
Nonsurgical Vastus Lateralis EI	108.13 (<0.001)	90.00, 126.92	0.22 (0.870)	−2.55, 2.63	−0.57 (0.270)	−1.63, 0.35
Surgical Rectus Femoris EI	107.11 (<0.001)	92.40, 121.67	1.43 (0.090)	−0.08, 2.99	−0.24 (0.430)	−0.82, 0.34
Nonsurgical Rectus Femoris EI	110.29 (<0.001)	97.54, 122.83	−1.72 (0.110)	−3.61, 0.29	0.29 (0.450)	−0.51, 1.00

**Table 2:** Results from the Pearson *r* correlation analyses for change scores between weeks 6 and 10. None of the correlations were statistically significant (*p* > 0.05).

Pearson’s Correlations			
Change in Each Variable Between 6 and 10 Weeks		Pearson’s *r*	*p*
IKDC	Peak torque (Nm/kg)	0.034	0.915
	Rectus Femoris CSA (cm^2^)	–0.262	0.411
	Rectus Femoris EI (A.U.)	0.230	0.472
	Vastus Lateralis CSA (cm^2^)	–0.570	0.053
	Vastus Lateralis EI (A.U.)	0.133	0.680
Peak Torque (Nm/kg)	Rectus Femoris CSA (cm^2^)	0.407	0.190
	Rectus Femoris EI (A.U.)	0.062	0.849
	Vastus Lateralis CSA (cm^2^)	0.220	0.493
	Vastus Lateralis EI (A.U.)	0.246	0.441
Rectus Femoris CSA (cm^2^)	Rectus Femoris EI (A.U.)	0.233	0.465
	Vastus Lateralis CSA (cm^2^)	–0.017	0.957
	Vastus Lateralis EI (A.U.)	0.266	0.402
Rectus Femoris EI (A.U.)	Vastus Lateralis CSA (cm^2^)	–0.277	0.384
	Vastus Lateralis EI (A.U.)	0.154	0.632
Vastus Lateralis CSA (cm^2^)	Vastus Lateralis EI (A.U.)	0.290	0.361

## Data Availability

Research data for this study are available by contacting the corresponding author.

## Supplementary Materials

The following supporting information can be downloaded at: https://www.mdpi.com/article/10.3390/jfmk11020200/s1.

## Abbreviations

The following abbreviations are used in this manuscript:

ACL	Anterior cruciate ligament
ACLR	Anterior cruciate ligament reconstruction
A.U.	Arbitrary units
BMI	Body mass index
cm	Centimeters
CSA	Cross-sectional area
dB	Decibels
EI	Echo intensity
Hz	Hertz
ICC	Intraclass correlation coefficient
IKDC	International Knee Documentation Committee
IRB	Institutional Review Board
MHz	Megahertz
MRI	Magnetic resonance imaging
N	Newtons
Nm	Newton-meters
REDCap	Research Electronic Data Capture
RF	Rectus femoris
SD	Standard deviation
STROBE	Strengthening the Reporting of Observational studies in Epidemiology
USA	United States of America
VL	Vastus lateralis
