# Supplementary material for: A Comparison of Methods for Tracking Muscle Quality During Early-Phase Rehabilitation Following Anterior Cruciate Ligament Reconstruction
Source: J Funct Morphol Kinesiol. 2026 May 17;11(2):200. doi: 10.3390/jfmk11020200 (PMC13214610; doi:10.3390/jfmk11020200)
Supplement: Supplementary file 1 [file jfmk-11-00200-s001.zip › jfmk-4083197-supplementary.pdf]

STROBE Statement—checklist of items that should be included in reports of observational studies

	Item No.	Recommendation	Page No.	Relevant text from manuscript
**Title and abstract**	1	(*a*) Indicate the study's design with a commonly used term in the title or the abstract	**1**	Using an observational, cohort design, we examined changes in quadriceps muscle strength, size, and quality, along with self-reported knee function, 2, 6, and/or 10 weeks following anterior cruciate ligament reconstruction (ACLR).
		(*b*) Provide in the abstract an informative and balanced summary of what was done and what was found	**1**	We conclude that quadriceps strength, size, quality, and self-reported knee function change independently and do not follow a shared recovery trajectory.
**Introduction**				
Background/rationale	2	Explain the scientific background and rationale for the investigation being reported	**2**	Despite growing interest in tracking quadriceps function following ACLR, key knowledge gaps remain. Comprehensive data on how quadriceps EI changes during rehabilitation and whether these changes align with muscle strength, size, or patient-reported function are lacking. Clarifying how these measures evolve can help clinicians refine and personalize treatment plans.

Objectives	3	State specific objectives, including any prespecified hypotheses	**2**	This study aimed to track changes in muscle strength (peak torque), size (CSA), quality (EI), and self-reported knee function following ACLR. Based on prior research, we hypothesized that strength, EI, and IKDC scores would improve but follow trajectories distinct from CSA.
**Methods**				
Study design	4	Present key elements of study design early in the paper	**3**	This study utilized a repeated measures design in individuals aged 15–40 years who underwent ACLR within 12 months of injury.
Setting	5	Describe the setting, locations, and relevant dates, including periods of recruitment, exposure, follow-up, and data collection	**3**	To enhance participant retention, testing was offered either at the University of Central Florida (Orlando, FL, USA) or at the participant's physical therapy clinic.
Participants	6	(*a*) *Cohort study*—Give the eligibility criteria, and the sources and methods of selection of participants. Describe methods of follow-up<br>*Case-control study*—Give the eligibility criteria, and the sources and methods of case ascertainment and control selection. Give the rationale for the choice of cases and controls<br>*Cross-sectional study*—Give the eligibility criteria, and the sources and methods of selection of participants	**3**	The study was designed to assess outcomes at 2, 6, and 10 weeks following ACLR…. All participants had undergone ACLR within the six weeks prior to enrollment. Before enrolling, participants completed a phone-based pre-enrollment health screening to

				ensure eligibility and minimize risk. Exclusion criteria included a BMI < 20 or > 35 kg/m², significant pathology or pain affecting the nonsurgical limb, bilateral ACLR, or limb amputation. Additional exclusions included current pregnancy, use of hormone therapy in the previous six months, history of intra-articular injections, cancer, stroke, or any metabolic, neuromuscular, or degenerative disease. English proficiency was required.
		(*b*) *Cohort study*—For matched studies, give matching criteria and number of exposed and unexposed<br>*Case-control study*—For matched studies, give matching criteria and the number of controls per case	**N/A**	N/A
Variables	7	Clearly define all outcomes, exposures, predictors, potential confounders, and effect modifiers. Give diagnostic criteria, if applicable	**N/A**	N/A
Data sources/ measurement	8*	For each variable of interest, give sources of data and details of methods of assessment (measurement). Describe comparability of assessment methods if there is more than one group	**3-5**	Methods section describes in great detail how each dependent variable was assessed; only one group was studied.
Bias	9	Describe any efforts to address potential sources of bias	**N/A**	N/A
Study size	10	Explain how the study size was arrived at	**3**	We recruited participants using a combination of convenience and snowball sampling, leveraging clinical partnerships with physical therapists to

promote the study. The final sample included 13 participants (9 females, 4 males), ranging from 16 to 35 years of age (mean ± SD: 23 ± 5 years) with a body mass index (BMI) of 25.2 ± 2.9 kg/m².

Continued on next page

Quantitative variables	11	Explain how quantitative variables were handled in the analyses. If applicable, describe which groupings were chosen and why	7-8	Continuous variables (peak torque, IKDC scores, CSA, corrected EI, age, and BMI) were analyzed as quantitative variables. Age and BMI were grand-mean centered by sex. Sex and graft type were entered as categorical predictors in multilevel models. No variables were categorized for primary analyses.
Statistical methods	12	(*a*) Describe all statistical methods, including those used to control for confounding	7-8	Two-level multilevel models using restricted maximum likelihood were performed with the lme4 package in R. Repeated observations were nested within participants. Sex, graft type, age, and BMI were included as covariates to account for potential confounding. Statistical significance was set at $p < 0.05$.
		(*b*) Describe any methods used to examine subgroups and interactions	7-8	Separate multilevel models were fit for each outcome and limb. No formal subgroup analyses or interaction terms beyond time effects were included.
		(*c*) Explain how missing data were addressed	7-8	Multilevel modeling with full information maximum likelihood allowed all available data to be included, even when participants missed one or more visits, under the assumption that data were missing

				at random.
		(*d*) *Cohort study*—If applicable, explain how loss to follow-up was addressed<br>*Case-control study*—If applicable, explain how matching of cases and controls was addressed<br>*Cross-sectional study*—If applicable, describe analytical methods taking account of sampling strategy	7-8	Of the 13 participants, 12 completed the 10-week visit. One participant completed only the 2- and 6-week visits. Multilevel models retained all available observations without requiring imputation.
		(*e*) Describe any sensitivity analyses	N/A	No formal sensitivity analyses were performed.
**Results**				
Participants	13*	(a) Report numbers of individuals at each stage of study—eg numbers potentially eligible, examined for eligibility, confirmed eligible, included in the study, completing follow-up, and analysed	4–5, 8	Thirteen participants were enrolled. One participant completed the 2- and 6-week visits only; six completed visits at weeks 6 and 10 only; and six completed all three visits.
		(b) Give reasons for non-participation at each stage	8	Reasons included unanticipated travel and learning of the study after the optional 2-week time point.
		(c) Consider use of a flow diagram	N/A	A participant flow diagram was not included.
Descriptive data	14*	(a) Give characteristics of study participants (eg demographic, clinical, social) and information on exposures and potential confounders	4-5	Demographic and clinical characteristics included age, sex, BMI, graft type, meniscal repair status, and postoperative management.
		(b) Indicate number of participants with missing data for each variable of interest	8	Missing data were due to incomplete attendance at some visits. Sample sizes varied by

				outcome (e.g., N = 31 repeated observations for most outcomes; N = 29 for peak torque).
		(c) *Cohort study*—Summarise follow-up time (eg, average and total amount)	3-4	Participants were assessed at approximately 2, 6, and 10 weeks after ACL reconstruction.
Outcome data	15*	*Cohort study*—Report numbers of outcome events or summary measures over time	8-12	Summary measures for peak torque, IKDC, CSA, and corrected EI are presented in Table 1, Table 2, and Figures 2–4.
		Case-control study—Report numbers in each exposure category, or summary measures of exposure		
		Cross-sectional study—Report numbers of outcome events or summary measures		
Main results	16	(*a*) Give unadjusted estimates and, if applicable, confounder-adjusted estimates and their precision (eg, 95% confidence interval). Make clear which confounders were adjusted for and why they were included	8-12	Regression coefficients, p values, and 95% confidence intervals are reported in Table 1. Models adjusted for sex, age, BMI, and graft type.
		(*b*) Report category boundaries when continuous variables were categorized	N/A	Continuous variables were not categorized.
		(*c*) If relevant, consider translating estimates of relative risk into absolute risk for a meaningful time period		

Continued on next page

Other analyses	17	Report other analyses done—eg analyses of subgroups and interactions, and sensitivity analyses
**Discussion**		
Key results	18	Summarise key results with reference to study objectives
Limitations	19	Discuss limitations of the study, taking into account sources of potential bias or imprecision. Discuss both direction and magnitude of any potential bias
Interpretation	20	Give a cautious overall interpretation of results considering objectives, limitations, multiplicity of analyses, results from similar studies, and other relevant evidence
Generalisability	21	Discuss the generalisability (external validity) of the study results
**Other information**		
Funding	22	Give the source of funding and the role of the funders for the present study and, if applicable, for the original study on which the present article is based

*Give information separately for cases and controls in case-control studies and, if applicable, for exposed and unexposed groups in cohort and cross-sectional studies.

Note: An Explanation and Elaboration article discusses each checklist item and gives methodological background and published examples of transparent reporting. The STROBE checklist is best used in conjunction with this article (freely available on the Web sites of PLoS Medicine at http://www.plosmedicine.org/, Annals of Internal Medicine at http://www.annals.org/, and Epidemiology at http://www.epidem.com/). Information on the STROBE Initiative is available at www.strobe-statement.org.
